# Recovery, Characterization, Functionality and Applications of Bioactive Compounds from Food-Plant Products and Their By-Products

**DOI:** 10.3390/plants12233964

**Published:** 2023-11-24

**Authors:** Ariel Fontana

**Affiliations:** Grupo de Bioquímica Vegetal, Instituto de Biología Agrícola de Mendoza CONICET-UNCuyo, Almirante Brown 500, Chacras de Coria M5528AHB, Argentina; arfontana82@gmail.com

## 1. Introduction

Sustainability in the food industry has been frequently linked to strategies that avoid, or at least minimize, the amount of by-products in food production. Although reducing these side streams is highly desired, the processing of plants and fruits is unavoidably associated with their occurrence. In the context of consumers increasingly taking interest in natural products without synthetic preservers, with labels, for instance, detailing a full breakdown of resources and with specific components providing additional health benefits, the recovery of bioactive components from food and plant industry by-products has acquired much interest.

Plant-derived phytochemicals have demonstrated several in vitro and in vivo biological effects. They have also been used as ingredients for food functionalization due to their health-promoting effects and their potential to improve the conservation of foodstuffs in a natural, current, and more tolerable way for certain segments of society. In most cases, these effects have been connected to the antioxidant properties of different families of bioactive compounds, such as phenolic compounds (PCs), carotenoids, tocopherols, phytosterols, and peptides, amongst others. As an important area of research, this centers on the exploration and identification of bioactive components from plant sources in order to clarify the correlation between the chemical composition and the functionality of natural ingredients. This topic plays a key role in both structure-guided bioactivity and interactions between component (synergic or antagonist) effects in a complex matrix such as those comprising plant extracts.

The extraction and characterization steps of these components are directly linked to the functionality and applicability of bioactive compounds, because, according to the conditions used for recovery, the composition and potential function of components can be altered (in positive or negative ways). Understanding the stability and functionality of isolated components, including their bioaccessibility and bioavailability, is necessary in order to establish and enable the application of phytochemical extracts as functional ingredients. Additionally, a compound, or families of compounds, may suffer changes during food elaboration, which can not only result in the modification of their bioavailability but also in the modification of their biological properties. In the same vein, changes in the food quality and acceptability of a product can be altered; therefore, an evaluation of chemical changes during storage is also necessary.

Consequently, this SI presents a compilation of 20 articles, including five reviews, where the contributing authors aim to deepen our understanding of certain specific aspects related to bioactive components as derived from the plant-based food industry. The topics delineated below undertake a variety of foci and plenty of matrices, in addition to findings concerning, for instance, the health-promoting applications of phytochemicals, as supported via chemical characterizations.

## 2. An Overview of the SI

In their contribution, Hammoud et al. [1] conducted a search for alternative, improved methods in bioactive compound recovery. The authors proposed the utilization of the Ired-Irrad^®^ (IR) technique to intensify the PC extraction of the medicinal plant Eryngium creticum, as IR uses the infrared energy produced by a ceramic emitter to heat solvent–matrix mixtures. The authors optimized the PC extraction of Eryngium creticum leaves with IR for the first time and compared the results with those of the conventional water extraction method. Additionally, different biological effects such as the antiradical, antioxidant, antibacterial, and antibiofilm activities of both extracts were assessed. Under optimal conditions, IR improved PC extraction yield by 1.7 times, whereas the consumption of ethanol was 1.5 times lower. Both of the IR- and water-based extracts exhibited the highest antibacterial activity against Staphylococcus epidermidis. Using ultra-high performance liquid chromatography–mass spectrometry (UHPLC-MS), the authors quantified two major PCs in both extracts: rutin and sinapic acid. In light of these results, IR technology was proven to be an effective option in the improvement of PC recovery.

In the same vein regarding alternatives to traditional solid–liquid extraction methods, a microwave-assisted extraction (MAE) method of PCs from organic peppermint (Mentha piperita L.) leaves was optimized [2]. In this study, the authors evaluated the influence of irradiation power on the extraction rate, finding that irradiation power exerted significant influence, whereas its influence on the asymptotic value of the response was negligible. These results are projected to enable the sustainable production of value-added, peppermint-based products, which would be designed for high-value applications in technological fields.

In another contribution, Kabbas Junior et al. [3] studied chemical and cellular antioxidant activity, cytotoxicity in human cells, and the peroxidative inhibition of PC extracts recovered from defatted grape (*Vitis labrusca* L.) seeds and blackberry (*Rubus fruticosus* L.) seeds. In this study, the authors demonstrated that grape and blackberry seed extracts did not induce ROS generation in two cancer cells lines. Furthermore, the extracts were able to protect against H_2_O_2_-induced ROS production, and they demonstrated both antioxidant activity and a protective effect on erythrocytes when subjected to hypotonic and isotonic conditions.

Another study detailed the application of less conventional analytical strategies for the fractionation of PCs, evaluated through the use of high-performance counter-current chromatography (HPCCC), on the by-product extracts of the wine industry [4]. After refining the ethanol–water extracts of grape pomace (GP) and wine lee (WL) by-products through an ethyl acetate extraction, the HPCCC system, which was composed of n-hexane–ethyl acetate–methanol–water (1:5:1:5), yielded an enriched fraction of a minor family of flavonols in these by-products. As a result, high recoveries of purified flavonols (myricetin, quercetin, isorhamnetin, and kaempferol) in GP and WL were obtained. In addition to these findings, concentration capabilities of the HPCCC system were examined with the use of UHPLC-MS for the study of the composition and a tentative identification of PCs in these matrices. As a result of this examination, a total of 57 PCs in both matrixes were identified, 12 of which were reported for the first time in WL and/or GP. These findings point to opportunities for the use of HPCCC as a convenient alternative for the obtention of an adequate amount of minor PCs, such as flavanols from GP and WL, for studies on the correlation among structures, synergies, and bioactivity in food systems.

Further regarding the exploration of winemaking industry by-products, Salem et al. [5] proposed a multi-step, biomass fractionation of seeds from grape pomace to obtain different potential value-added products. The first step consisted of the recovery of lipids from seeds obtained from two traditional grape varieties in Lebanon, with the authors reporting yields higher than 12% (*w*/*w*). The second step consisted of PC recovery from the defatted seeds. In the third step, the defatted and dephenolized seeds were extracted under alkaline conditions, and the proteins were recovered via precipitation at an isoelectric point, with yields of 4% (*w*/*w*). The proposed approach is an interesting alternative to existent methods concerning the reduction in side streams in the wine industry.

Nouioura et al. [6] formulated antioxidants from the following three plants grown in Northern Morocco: *Apium graveolens* L., *Coriandrum sativum* L., and *Petroselinum crispum* M. The antioxidant properties of the optimized combination offered better results than the use of the plant extracts individually, reinforcing the view that the potential use of plant combinations results in higher antioxidant activities and beneficial effects than their use individually, as can be seen in this case in particular.

As previously mentioned, bioactive compounds may suffer changes during storage and food processing, and these can be related to a loss in quality and/or claimed bioactive properties. In another contribution, Monasterio et al. [7] studied the evolution of quality parameters and chemical changes (PCs and triterpenic compounds) in virgin olive oil (of the Arauco variety) under different storage conditions. These authors evaluated the effects of exposure to light, temperature, packaging material, and headspace during a period of 76 days. Apart from the samples maintained in compliance with EFSA health claims after different storage conditions, a reduction in the total number of PCs was observed after treatment. The results revealed that the preservation of oil in PET appeared to be an adequate treatment, with improved stability when N2 was used in the headspace, along with darkness and lower temperatures. A study of phenolic profiles revealed that compounds previously reported as possible markers of olive oil aging continued to maintain a similar performance during the aging of Arauco variety oil. Remarkably, evidence was presented that another oleuropein-derived compound (oleuropein aglycone isomer 3) could also be used as an aging marker.

Tuárez-García et al. [8] studied the effects of different heating treatments (boiling, roasting, and baking) on the antioxidant activity and PC profiles of Ecuadorian Red Dacca bananas. By using UHPLC–high resolution (HR) MS, a total of 68 PCs were identified, or tentatively identified, in raw bananas and in treated samples, with flavonoid content (flavan-3-ols with 88.33% and flavonols with 3.24%) highlighted in raw banana samples in particular. In all of the cooking conditions evaluated, PC content decreased, as the flavan-3-ols and flavonols groups were those most affected by the heating treatments. In addition, antioxidant activity also decreased as a result of the decrease in these PCs.

Present definitions in the collection of samples and the establishment of concentrations of phytochemicals is important for the standardization of extracts, especially when they are intended to be regularly used as additives in a particular system. With the aim of classifying olive leaf extracts based on variety and season, a metabolomics approach was proposed in order to study the PCs in this matrix [9]. The authors applied LC–ORBITRAP–MS to identify PCs in olive leaf extracts from five varieties grown in the Apulia region (Italy) during two different seasonal times, concluding, from their results, that the leaves collected had more PCs and antioxidant activities.

Another work in this SI focused on the characterization of medicinal plants through the chemical and antioxidant characterization of essential oils (EOs) from the Lamiaceae (or mint family), Asteraceae, Cupressaceae, and Lamiaceae families grown in Serbia [10]. Hydrodistillation (HD) was used for the isolation of the EOs, with yield and terpenoid profiles evaluated via gas chromatography (GC)–MS. The results presented in this contribution indicated distinctions among the selected plant species based on terpenoid composition and antioxidant activities. These data could be implemented in technological applications as a potential screening tool in the selection of EO candidates for extract-obtaining purposes.

Another group of bioactive compounds evaluated in this SI was isoflavones. Arora et al. studied the total isoflavone content in chickpea (*Cicer arietinum* L.) sprouts, germinated under precursors such as p-coumaric acid and L-phenylalanine [11]. The authors observed that an increase in sprouting time for a period up to seven days resulted in isoflavone content that was approximately eight times higher than that in chickpea seeds. After the third day of p-coumaric acid supplementation, an increase of more than 150% was observed in the PCs. Additionally, the increase in TPC was positively correlated with the antioxidant activity observed. This study suggests that chickpea sprouts enriched in TPC and antioxidant compounds can be produced by managing the amount of precursor supplementation. In addition to this, the authors also highlighted findings indicating that the consumption of 100 g of seventh-day sprouts provided isoflavones with a yield that was eight times higher in comparison to chickpea seeds.

Texeira et al. [12] studied the effects of two novel fruit juice production alternatives, namely centrifugal decanter and tangential filtration, on the PC profile of juices and by-products that remained after juice elaboration. The objective was to propose ‘Not from Concentrate’ (NFC) pear and apple juice options to consumers and evaluate the effects of these technologies on PC composition. The authors concluded that, despite the knowledge that turbid juices have more PCs than clarified juices, the use of tangential filtration with low temperatures for clarification can result in juices with similar PC content to those of the turbid juices produced using centrifugal decanters at high differential speeds. In fact, NFC juices produced using centrifugal decanters and tangential filtrations are appropriate alternatives in the obtention of juices with a higher amount of PCs.

A contribution by Cruz-Carrión et al. [13] focused on present PC composition in organic (ORG) and non-organic (NORG) plant-based foods. They analyzed the antioxidant activity and PCs of several plant-based foods cultivated in ORG and NORG systems. The results from the pool of samples analyzed demonstrated that NORG fruits had a tendency to contain higher levels of PC content, whereas ORG fruits had more antioxidant capacities. In general, there was no clear, general pattern of differentiation. In fact, the authors suggested that the effects of the type of cultivation tended to depend on the plant species studied. Consequently, future studies should analyze samples cultivated in the same place and under the same ORG and NORG exposure conditions, with an emphasis placed on the further exploration of potential differentiation. 

In terms of the functionality of bioactive components, this SI reported several interesting applications, with one of them being focused on proteins and peptides (the only paper in this Issue containing these bioactive compounds) derived from a soy protein isolate [14]. Soybean proteins and peptides, such as lunasin, have been considered promising bioactive compounds in the regulation of inflammatory and oxidative processes and mediators, such as reactive oxygen species (ROS) and nitric oxide (NO). With this in mind, the authors studied the antioxidant and immunomodulatory properties of a soluble soybean protein isolate and its fractions. The fractionation process through ultrafiltration achieved an enrichment of the multifunctional peptide lunasin, in addition to many identified sequences, which could have contributed to the effects observed as well. A protective effect against the oxidative stress induced by LPS in the macrophage model could be mediated by the radical scavenging capacity of the peptides present in the soybean samples. Beyond these results, the identification of the major contributors responsible for health benefits and a complete elucidation of the mechanisms of action are needed for the final confirmation of the promising role of soybean proteins/peptides as ingredients in functional foods or nutraceuticals.

Regarding functionality in this context, an investigation by Lela et al. undertook an original evaluation of the antilipidemic and anti-inflammatory activity of the Lucanian *S. aethiopicum* L. peel extract in vitro on oleic acid (OA)-treated HepG2 and Caco-2 cell lines [15]. This extract was able to reduce fat accumulation by promoting lipolysis and lipid elimination instead of lipogenesis. Additionally, this *S. aethiopicum* L. peel extract was hypothesized to potentially improve oxidative stress by reducing endoplasmic reticulum stress. Based on this evidence, the Lucanian *S. aethiopicum* peel extract could be a promising source of active molecules in the prevention of obesity.

The bacteriostatic and preservative effects of extractable condensed tannins (ECTs), as isolates from longan pericarps and seeds, was evaluated by Wang et al. [16] These pericarps and seeds are considered by-products of longan production and processing with an abundance of PCs. For the purposes of their work, the authors recovered the PCs, particularly the ECTs—and observed that the antioxidant capacity of the ECTs from the longan pericarps and seeds was more than 60% higher than those measured in fresh samples. A subsequent exploration of antimicrobial activity revealed that the ECTs had significant inhibitory effects on *Pseudomonas aeruginosa*, *Escherichia coli*, Salmonella, and *Staphylococcus aureus*. Moreover, regarding the preservative effect of the extracts applied to fresh-cut lotus roots, the following effects were observed after ECT treatment: the browning degree was reduced, the color was better maintained, the respiration was inhibited, the nutrient loss was reduced, the bacterial reproduction was inhibited, and the cell senescence was slowed. All of these results demonstrably indicate that ECTs from the by-products of dried longan pericarps and seeds could be used as natural preservatives for fresh-cut fruits and vegetables.

In this SI, several reviews comprising different topics in terms of bioactive compounds, sources of origin, and functionalities were also published. One of these reviews offers an overview of sources and techniques used for the sustainable isolation of bioactive substances and proteins resulting from various sources that generate waste in the preparation or production of food of plant origins [17]. This review covers different approaches for the use of waste and by-products from the food industry, with the aim of maximizing the benefits of their use. Silvano Arruda et al. [18] presented a scientific compilation of work in the last decade on the recovery, characterization, and functionality of bioactive compounds from the Araticum Fruit (Annona crassiflora Mart.), which is a fruit from a native and endemic plant in the Brazilian Cerrado region and has high sensorial, nutritional, bioactive, and economic potential, with its main use occurring in local folk medicine. The authors compiled data indicating that Araticum fruit parts contain several families of bioactive compounds, including PCs, alkaloids, annonaceous acetogenins, carotenoids, phytosterols, and tocols. These phytochemicals also feature in various biological activities, but further studies—particularly toxicological, pre-clinical, and clinical trials—must be conducted in order to confirm biological effects in humans, as well as evidence of their safety for consumers. In another review, Kumaro et al. [19] compiled various bioactive compounds isolated from *Acacia catechu* (L.f.) Willd. plant tissues and delineated their health-promoting functionalities. As a member of the family Fabaceae (subfamily Mimosoideae), known as Khair or Cutch tree, this plant possesses diverse pharmacological capabilities. This review aims to highlight the phytochemical profile of different parts of A. catechu, the different biological activities of the A. catechu extract, and its potential utilization in foods and beverages. Lastly, a review of chlorophylls as a natural bioactive compound present in food by-products focused on providing insight into the properties of chlorophylls and the effect of different treatments on their stability [20]. In addition, the most recent chlorophylls extraction approaches, from a sustainable perspective, are highlighted in this review.

## 3. Conclusions

The articles included in this SI describe the extraction, chemical characterization, and functionalities of different bioactive compounds recovered from plant-based food products and their by-products. The families of the bioactive compounds covered in this SI include PCs, terpenoids, chlorophylls, alkaloids, annonaceous acetogenins, carotenoids, phytosterols, tocols, triterpenic compounds, proteins, and peptides. Most of the contributions to this SI demonstrate associations between the composition of bioactive compounds and the antioxidant activities of extracts and/or isolated fractions, with contributions delineating their antimicrobial, preservative, anticancer, antilipidemic, anti-inflammatory, and immunomodulatory properties, among others. These properties are key factors and must be addressed whenever extracts or purified compounds are proposed as potential functional phytochemicals. Consequently, most of the papers are in agreement regarding the correlation of the concentration or presence of different compounds with the bioactivities reported; as a result, the importance of these connections in the composition of extracts is underlined. The objective of this SI is to provide readers with a wide scope of the functionalities and backgrounds of the compounds responsible for said connections, with special emphasis placed on the importance of the safety and value of natural resources.

## Data Availability

All the data is available in the papers published in this special issue.
